# Beyond Components of Wellbeing: The Effects of Relational and Situated Assemblage

**DOI:** 10.1007/s11245-013-9164-0

**Published:** 2013-05-17

**Authors:** Sarah Atkinson

**Affiliations:** Centre for Medical Humanities, c/o Department of Geography, Durham University, Durham, DH1 3LE UK

**Keywords:** Wellbeing, Components, Situated, Relational, Performance, Process

## Abstract

Despite multiple axes of variation in defining wellbeing, the paper argues for the dominance of a ‘components approach’ in current research and practice. This approach builds on a well-established tradition within the social sciences of attending to categories whether for their identification, their value or their meanings and political resonance. The paper critiques the components approach and explores how to move beyond it towards conceptually integrating the various categories and dimensions through a relational and situated account of wellbeing. Drawing on more fluid social sciences, wellbeing is framed as an effect, dependent on the mobilisation of resources from everyday encounters with complex assemblages of people, things and places. Through such a framing, wellbeing can be conceived of as stable and amenable to change, as individual and collective and as subjective and objective. Policy interventions then need to attend to the relationalities of particular social and spatial contexts.

## Introduction

Most people have pondered at some point in their lives what it is that constitutes a good and flourishing life. Despite an illusion of simplicity, the responses to this one question are legion. For centuries, response has galvanised political contestation, inspired creative endeavours, enabled the profitable commoditisation of desires and, of course, nourished philosophical enquiry. These areas of response, and there are many more that could be listed, start to hint at the multiplicity of influences that can give equally multiple meanings to what is a good and flourishing life. And whilst Aristotle is often credited with first interrogating both the question and the response, each age has witnessed differently framed engagements. Our contemporary engagement in the twenty-first century is manifest and expressed through the concept of wellbeing. The word is everywhere: in exhortations for individual action (Rath and Harter 2010), in marketing goods and services (Kim and Cho 2012; Little 2012), in good employer criteria (Robertson and Cooper 2011) and in a repositioning of the goals of government and policy intervention (nef 2004; Stiglitz et al. 2009). There is increasing momentum and spread of a move away from equating doing well as a government with ensuring economic security, growth and the material prosperity of the population. This move is premised on an argument that although economic status has a close association with health and wellbeing, economic performance should be seen as part of the means to human flourishing, not the end itself (Sen 1999). The publication at the end of September, 2009, of the high profile Report by the Commission on the Measurement of Economic Performance and Social Progress, chaired by Stiglitz et al. (2009), effectively reconceptualised a flourishing society as much more than economic production and explored ways for performance measures to include indicators of wellbeing, including people’s own subjective and personal assessments. Similar initiatives are to be found in Canada and most recently in the United Kingdom where, in 2011, the British government commissioned the Office for National Statistics to consult and develop measurements of subjective wellbeing as part of assessing social progress (Self et al. 2012). However, wellbeing is a notoriously abstract and unstable term. The move towards wellbeing in policy includes recognition that wellbeing is a complex and multidimensional concept, but as a complex and multidimensional concept, there are various alternative ways of defining, interpreting and mobilising wellbeing for policy purposes. It is incumbent upon academia to reflect not only on the abstract philosophical aspects to wellbeing, but also to reflect on which meanings are actually favoured in practice, particularly by government and policy communities, to unpick the assumptions that underpin current mobilisations and thereby to foster a critical engagement with the current enthusiasm for wellbeing.

The paper will first lay out an argument that, despite much talk over the lack of conceptual clarity in the term wellbeing, a dominant usage is evident which has several characteristics that frame, direct and constrain the actions and interventions under consideration as options for enhancing wellbeing. The second section will then engage critically with this dominant usage to draw out the ways this both fits and frames an individualised responsibility for our own wellbeing. Finally, the paper proposes an alternative framing in which wellbeing is always and necessarily situated and relational, an effect of mutually constitutive interactions amongst the material, organic and emotional dynamics of places. Shifting the focus from individual acquisition and foregrounding relationality and place offers new ways to understand wellbeing, move beyond the categories and boundaries of contemporary neoliberal policy and explore alternative modes of action and intervention in advancing human flourishing.

## A Dominant Framing of Wellbeing

Policy-facing research on wellbeing can been seen as most often embedded within an unchallenged line of argument that follows its logic through three points (see for example, Allin 2007; McGillivray and Clarke 2006; Diener et al. 2009). First, there is a lack of agreement on terminology, definitions and monitoring tools; secondly, this is important because different understandings and definitions of wellbeing risk creating barriers to communication across different sectors involved in policy-making; thirdly, in order to evaluate the benefits of different policy interventions in terms of enhancing wellbeing, there is a need for standardised indicators and monitoring tools. The case for greater attention to wellbeing has emerged through movements related to various political concerns including those addressing broad development goals, environmentally sustainable living, and a focus on an individualized, psychological state of happiness or flourishing. Whilst the concept of wellbeing unifies these different calls for policy to look beyond economic measures of social progress, the different engagements can be distinguished across various dimensions including those of scale, scope, location and responsibility. Several discourses of wellbeing are documented as co-existing within current policy debates, and, moreover, the specifics of how wellbeing is defined through particular aspects and how these may be weighted in different approaches show great variation (Ereaut and Whiting 2008; Atkinson and Joyce 2011). However, this evident variation notwithstanding, there is also a considerable degree of convergence in the underlying contemporary approach to wellbeing that informs both policy documents and policy-facing research.

Research and policy mostly deal with the abstract nature of wellbeing by breaking it down into constitutive dimensions in what has been called ‘a components approach to wellbeing’ (Atkinson and Joyce 2011; Atkinson et al. 2012). In this approach, debate centres on the identification and theorisation of the independent elements that comprise wellbeing. Some schemas, often associated with assessing social progress of collective units, identify a mix of objective and subjective elements (for example, Clarke 2006; Nussbaum 2000; Stiglitz et al. 2009). Other schemas elaborate the components of personal subjective wellbeing, typically differentiated by hedonic (for example, Layard 2005; Seligman 2011) and eudaimonic variants (for example, Ryff 1989; Ryff et al. 2004; Veenhoven 2000; see also Deci and Ryan 2008; Ryan and Deci 2001). Component lists have also been derived from empirical research on people’s own definitions of what is important to their wellbeing; the United Kingdom’s Office for National Statistics (Self et al. 2012) shaped a national consultation on wellbeing through a set of domains derived from preliminary public engagement (relationships, health, activities of work/leisure and balance, where we live, personal finance, education and skills, contextual domains of governance, economy and natural environment). Depending on the entry point into exploring wellbeing, the factors that commonly appear in this kind of debate may be cast as either components or determinants of wellbeing. So in research on personal subjective wellbeing, the economic and a range of other social elements are cast as determinants, as in the influential Easterlin paradox (Easterlin 1974, 1995; Layard 2005; for a critique see Albor 2009; Stevenson and Wolfers 2008). For those attempting to define the entirety of human flourishing, independent dimensions to wellbeing cover a wide range of factors which both interact and respond to other external and collective determinants (Stiglitz et al. 2009).

A second feature of contemporary approaches to wellbeing is that they all share a common understanding of wellbeing as a quality that inheres to the individual. Wellbeing may be influenced by factors and processes from the individual to the global in scale and reach; it may be an objective characteristic or a subjective assessment; it may refer to a current state or a projection into the future, but the concept of well-being itself is individual in scale. Others have documented the emergence of the term wellbeing as predominantly individualised (Sointu 2005) and others have also pointed out that despite the individualisation of wellbeing, there are still alternative discourses mobilised that treat the concept as more collective, most prominently in relation to sustainability and environment (Ereaut and Whiting 2008; Atkinson and Joyce 2011). However, the argument here goes slightly further to claim that this individualisation of wellbeing also constitutes wellbeing as some kind of commodity, an entity that can be acquired, or at least achieved. And this is important in terms of policy as it drives intervention in terms of what can be done to enhance individual-directed acquisition of the components of wellbeing. A third feature of contemporary engagements with wellbeing is that one discourse of wellbeing has become dominant in research and in policy over others, that is, a discourse in which wellbeing is used as a synonym for health (Ereaut and Whiting 2008). For example, research through document analysis and interviews on the understanding of wellbeing within the 5 year strategies of local government authorities in the United Kingdom disclosed a striking tendency to conflate wellbeing with health (Atkinson and Joyce 2011). Moreover, this conflation goes even further in that not only is wellbeing reduced to health, but particularly to subjective or psychological health variously expressed through mental health, resilience or happiness (Layard 2005; Riva and Curtis 2012; Seligman 2011).

## Wellbeing, Outcome and Process

Conceptualising wellbeing as a set of entities that can be individually acquired does have value for both research and policy. A definition based on individually acquired entities allows us to ascribe stability to personal wellbeing, at least in the medium-term, and ascribing stability in turn enables the measurement of wellbeing through which to identify trends and associations and to evaluate interventions. Change in the acquisition of the components of personal wellbeing can be evaluated through the standard approach of a before and after assessment design. Building on a components approach, research and policy documents mostly position wellbeing as the desired outcome, assessing the relative influences of different determinants and trying to identify successful policy interventions to enhance that outcome. However, wellbeing can also be positioned as part of a process towards other desirable goals or criteria of success. The implications of thinking about wellbeing not only as a possibly desirable endpoint but also as a significant process factor can take at least three directions.

Thinking of wellbeing as process may challenge the contemporary dominant approach to wellbeing, specifically making a case against calls for a tighter definition of the concept. A study of how wellbeing was mobilised, understood and positioned within local government policy in the United Kingdom concluded that there are advantages to an ill-defined, somewhat abstract understanding of wellbeing. Rather than feeling frustrated at the lack of consensus on the definition of wellbeing, local government officials found that the various discourses of wellbeing in circulation provoked valuable reflection and discussion on the goals of local government action. The ill-defined nature of wellbeing also provided a concept through which highly divergent perspectives on important goals for local government could emerge in consultation with local partner community-based organisations (Atkinson and Joyce 2011). A relatively open understanding of wellbeing even brings advantages for the health sector. Although an understanding of wellbeing as synonymous with health might seem advantageous for medical expertise and medical concerns to take a central role in shaping and evaluating policy, in practice containing and controlling the concept within a health domain would undermine the opportunity offered by a broad understanding of wellbeing to build the intersectoral partnerships that are vital to advance a model of health that addresses a wide range of influences on differential health experiences (Atkinson 2011). Theoretical analyses of policy processes generally have similarly observed the value of key policy concepts that retain an ill-defined quality in enabling rhetoric, debate and flexibility in practice (Hajer 1995, 2005; Hajer and Versteeg 2005). Thus, wellbeing, exactly because of its vague and all-embracing character, offers a conceptual unifier across different sectors through acting as part of the process of policy-making as much as an outcome of policy-making. From this perspective, establishing a tight and measurable definition of wellbeing in order to evaluate outcomes would seriously limit the scope of policy work that the concept has the potential to achieve.

Thinking of wellbeing as process also exposes a separation of subjective wellbeing from more objective material aspects and health status as nonsensical, perhaps even dangerously illusionary. An argument that has attracted much attention in recent years is that inequalities in health relate strongly to relative inequalities in the distribution of material and developmental resources (Wilkinson and Pickett 2009). This association is strongly suggestive of a mediating role for a experiential happiness with one’s circumstances that is first and foremost situated in comparison with the norms of one’s social settings and that then impacts on other outcomes, particularly in this case health status. In this, it is difficult to distinguish which of happiness, health and wealth is the process and which the outcome. There is also a popular distrust of apparently uncritical happiness, a suspicion that enduring happiness may be founded on various cognitive and affective states that are considered deficient. In the context of Wilkinson’s relative inequalities thesis, for those with advantages, the ‘haves’, contentment and happiness may be based on naivety or ignorance about global and local relations of inequality and perhaps worse, complacency and a lack of care for the circumstances of others. For the ‘have-nots’, contentment and happiness may be based on a lack of aspiration and an acceptance of social injustices. Again, happiness is not only the outcome in these scenarios. In both cases, happiness and contentment not only reflect but also feed a lack of critical reflection on society’s values or a lack of imagination with respect to alternative possibilities.

In contrast, thinking of wellbeing as process may also respond to calls to fix the concept of wellbeing and narrow attention onto a personalised subjective understanding of wellbeing. Increasingly, both in research and in popular imagination, wellbeing is equated at an individual scale with various terms of positive affect. Happiness has tended to be the term to capture this partly perhaps because of its populist currency and partly because it can be slotted into an economist way of thinking as to what it is that people want to maximise in their lives, what drives their decision-making choices and how this can be enhanced through policy (Layard 2005). However, whereas an economic approach to wellbeing as happiness treats positive affect as the endpoint, the driver of other choices and the desired outcome of social policy, psychological approaches to wellbeing as happiness enable wellbeing to be attended to as both outcome and determinant of other outcomes. There is a rapidly growing body of research exploring the determinants of subjective wellbeing, happiness or other variants of positive affect, across a wide range of factors, but there is an equally rapidly growing literature treating subjective wellbeing or happiness as primarily under personal and internal control. In this work, wellbeing becomes no longer primarily an outcome of external factors, but a process of internal management and the object of personal responsibility. Moreover, this emphasis on personal action effectively positions many of the components typically comprising psychological definitions of subjective wellbeing as influences on various other personal outcomes and socially recognised criteria of success. These include many health conditions and health-related behaviours (Huber et al. 2011; Salovey et al. 2000), employment and earning capacity (de Neve and Oswald 2012), productivity at work (Robertson and Cooper 2010) and so on. The promise of the power of working on one’s own internal wellbeing, happiness or positive affect is reflected in an explosion of books grounded in the positive psychology movement that provide instruction on improving happiness in one’s life. The titles of publications disclose the emergence of this projection of subjective wellbeing as a self-directed approach. Indicative titles include the early texts from the founders of the positive psychology movement that promote procedures to acquire wellbeing, ‘*Learned optimism: how to change your mind*’ (Seligman 1990) and ‘*Flow: The psychology of optimal experience’* with a recent new cover describing it as ‘the classic work on how to achieve happiness’ (Csikszentmihalyi 1991/2002). More recent examples indicate an explicitly skill-oriented uptake of the message, such as ‘*Happiness: a guide to developing life’s most important skill*’ (Ricard 2003) and explicit reference to the benefits of positive affect for other areas of success in life, such as ‘*Positivity. Groundbreaking research to release your inner optimist and thrive’* (Fredrickson 2009) or ‘*The happiness advantage. The seven principles of positive psychology that fuel success and performance at work’* (Achor 2010).

Whilst there is undoubtedly evidence to support associations between positive psychology and other measures of success or resilience to adversity, an exclusive focus on wellbeing as internal and amenable to self-management logically leads to policy responses that similarly focus primarily on individual deficits in fostering and sustaining positive wellbeing. For example, cognitive behavioural therapy has gained a rapid momentum of uptake across a range of conditions and social contexts (see for example, Lloyd et al. 2012; Lynch et al. 2010; Mongia and Hechtman 2012). This approach, then, is becoming ever more dominant in wellbeing and happiness literature, but perhaps is best known through Seligman’s work on positive psychology, authentic happiness (2002) and more recently flourishing (2011). While wellbeing economists, such as Layard (2005), argue for a driving purpose to our choices and goals that can be captured through a notion of happiness, Seligman increasingly gives attention to what we ourselves can do to enhance our own wellbeing or flourishing. Seligman’s most recent elaboration posits five components, or ‘pillars’, of wellbeing captured by the acronym ‘PERMA’: positive emotion; engagement and interest (curiosity about the world); relationships; meaning; accomplishment (2011). The claim is that if we can achieve these qualities and if we can feed these qualities then we will feel better about how our lives are going, and indeed Seligman provides many positive experiences of such improvement.

There is an emergent literature beginning to counter some of the claims made for the power of subjective wellbeing, such as claims for beneficial effects on the outcomes of serious illness such as cancer (Coyne and Tennen 2010; Ehrenreich 2009). Research grounded in critical social and cultural theory also offer a challenge to this internalised and universalised presentation of wellbeing. The main critique levelled at this approach to wellbeing is that achieving these qualities is presented as largely an individual and internalised task of self-management, and that consequently failure of wellbeing can be positioned as failure of responsible citizenship. An individualised responsibility for such self-actualisation has been positioned as a significant feature of western society within a Foucauldian inspired analysis of contemporary forms of governance (Foucault 1991; Miller and Rose 2008). An analysis of the roots and development of the concept of wellbeing into its current usage details how in the 1960s and 1970s, wellbeing was understood as a collective concept, most commonly used in the context of the economy (Sointu 2005) or the moral attitudes to social and environmental inequalities (Smith 2000). Sointu (2005) explicitly locates the observable change in the dominant understanding and usage of the term wellbeing within the Foucauldian analysis of changes taking place over the same time period in dominant political ideology, and most particular those changes associated with the emergence of contemporary forms of neoliberalism. Seen through this lens, the question presents itself, both in relation to wellbeing as happiness and wellbeing as resilience, as to whether these are in fact outcomes or are rather the processes through which our conduct is directed according to the requirements of the political or economic imperatives of others.

The cultural theorist, Ahmed, elaborates this question further and profoundly problematises the very notion of happiness within contemporary western society, arguing that happiness is a highly politically charged concept. The avowed sources of happiness are pre-defined for us: for example, love, marriage and children; wealth, success and social standing; health, fitness and particular style. These constitute packages offered as promised routes to the good life and, as such, direct us towards making specific life choices which have considerable political content in reproducing social norms, consumption norms and various forms of discrimination (Ahmed 2010). This critique exposes a circularity in the argument of the positive psychology movement for individually targeted support for self-actualisation in that both the acquisition of personal subjective wellbeing and the acquisition of the associated elements of a good life are all normative values to which we are directed by our social situation, rather than any internal, decontextualized and ‘authentic’ notion of happiness. The acquisition of subjective wellbeing is then itself part of the definition of a successful and modern social self. Given the message that achieving success in all areas of life is apparently only limited by our own positive attitudes and choices, part of presenting oneself as a successful and modern social being necessarily involves enacting or performing an appearance of subjective wellbeing that may be at odds with any real state of positive affect. As such, wellbeing is clearly process rather than the outcome, a process through which to successfully perform self and negotiate inter-subjectivity. In an account of the emergence and imperative to demonstrate successful wellbeing in the context of the United States, Ehrenreich (2009) documents and critiques the currency of assertions that positivity has dominion over a range of material aspects of life, including wealth and physical health. An argument that the acquisition of those qualities defined as constituting a successful life is largely determined by internal attitudes is an argument for what many consider the illusion of a meritocratic society in which inequality can be dismissed through blaming the victims. Understanding that wellbeing may increasingly be performance rather than experience raises questions for how measuring wellbeing as an outcome informs policy purposes and what it can really tell us. Expressions of wellbeing are likely to be highly mediated in ways that vary in different specific social and spatial contexts.

## Wellbeing, Situated and Relational

The role of context in relation to affect is largely neglected within positive psychology, but is given very different treatment within cultural studies that examine the “affective colourings of socio-spatial life” (Anderson 2004: 740). To date this treatment has been little reflected within mainstream work on wellbeing or happiness. While there is a multiplicity of definitions and approaches to understanding affect, all share a decentring of the individual such that, “…there is no such thing as a pre-existing human subject who then encounters human or non-human others and emotes… All subjects are constantly constituted performatively, in encounters with other things.” (Rose et al. 2010: 345). Moreover, the very distinctions of subject and world, the inside and outside are themselves constituted through the circulation of affects (Anderson 2006). However, this emphasis on transpersonal capacity and the circulation and distribution of affects also requires attention to the unevenness in the flows of affects, of how they may cohere as a generative force, endure or dissipate, that is the need to attend to geopolitical landscapes, to power geometries, to historicity and, “the political fact of different bodies having different affective capacities.” (Tolia-Kelly 2006: 213). Exploring the unevenness in the circulation of affect has been taken up by Ahmed (2004a, b) who asks why and how affects ‘stick’ to certain signs rather than others. Ahmed provides an explicitly historicised account of affect; the temporality of affects is foregrounded as important not because of their fleeting quality as in other literatures, but because some affects are likely to become fixed to some signs, bodies or objects for different durations, and thus inform an affective politics. Ahmed’s particular attention to collective affects offers a very different lens through which to think about wellbeing. Here, the very boundaries of bodies, subjects and organisational structures are contoured by affective clusterings or settlements and constitute temporary fixing of nodal points. The distinction is dissolved between approaches in which the external world primarily affects the emotional responses and approaches in which the affective state of the subject primarily determines and interprets the external stimuli. Instead, “emotions work to create the very distinction between the inside and the outside, and that this separation takes place through the very movement engendered by responding to other and objects” (2004a: 28).

The cultural studies approach to affect and wellbeing offers an intriguing potential for working through the connections or blurring the distinctions between internal and external determinants of wellbeing, objective and subjective aspects of wellbeing and individual and collective levels of wellbeing. By focussing on the movement and clusterings of affect, wellbeing becomes a quality of situatedness and of relationality. However, many social theorists are not ready to let go fully of a notion of the centrality of individuals in social analysis, nor of wellbeing as inseparably interwoven with understandings of identity. Whether wellbeing is best conceptualised as outcome, as process or as a flow of affects is likely to depend on the focus of interest. Obviously much of the time it is useful to position some variant of wellbeing as the desired outcome of various interventions. The mainstream components approach in which wellbeing is constituted through a set of entities that can be individually acquired or achieved has value in that this ascribes stability to personal wellbeing which enables measurement, at least in the short- to medium-term and the evaluation of change. However, understanding change, and the processes through which intervention may engender change, requires an understanding of wellbeing as simultaneously unstable or able to be destabilised. Wellbeing thus needs to be understood not as sets of entities to be acquired as internalised qualities of individuals but instead as a set of effects produced in specific times and places (Kesby 2007).

Building on an understanding of wellbeing a both situated and relational informs the ‘spaces of wellbeing’ approach which offers a framing of how different social and spatial contexts may be facilitative (Fleuret and Atkinson 2007; Hall 2010). The framing allows wellbeing to be emergent through situated and relational effects that are dependent on the mobilisation of resources within different social and spatial contexts, thus bridging the typical divisions between individual and collective and subjective and objective. The approach proposes four interrelated spaces of resource mobilisation: capabilities (Nussbaum 2000), social integration (Putnam 2001; Wilkinson and Marmot 2003), security (Shaw 2004) and therapeutic processes (Conradson 2005; Smyth 2005). Wellbeing, as a set of situated and relational effects renders the embodied sense of self as deeply embedded within wider systems of recognition and misrecognition (Block and Kissell 2001; Fisher 2008; Prilleltensky 2005). Instead of social relations as something to acquire, a network of connections, the focus here is on the relationality itself. Moreover, wellbeing here comprises complex assemblages of relations not only between people, but also between people and places, material objects and less material constituents of places including atmosphere, histories and values (Panelli and Tipa 2009). Wellbeing is thus conceptualised as in constant production and reproduction. However, the habituated routines of everyday life, our situated repertoires of practice (Gutiérrez and Rogoff 2003), tend to reproduce, rather than destabilise, our embodied and embedded selves, which allows wellbeing to become stable and meaningful to measure over the medium-term. At the same time, this approach to wellbeing also offers a means to understanding how wellbeing can change both for better and worse and how interventions may facilitate destabilisations of habituated practices that open new relational assemblages. Framing wellbeing as relational and situated makes explicit that wellbeing can have no form, expression or enhancement without attention to the spatial dynamics of such effects. This alternative framing of wellbeing has associated alternative implications for how policy for wellbeing constitutes the primary focus of its interventions. A shift is demanded away from how to enhance the resources for wellbeing centred on individual acquisition and towards attending to the social, material and spatially situated relationships through which individual and collective wellbeing are effected. As Haidt proposes, ‘Happiness comes from the between’ (2006: 213).

## References

[CR1] Achor S (2010). The happiness advantage. The seven principles of positive psychology that fuel success and performance at work.

[CR2] Ahmed S (2004). Collective feelings: OR, the impression left by others. Theory Culture Soc.

[CR3] Ahmed S (2004). Affective economies. Soc Text.

[CR4] Ahmed S (2010). The promise of happiness.

[CR5] Albor C (2009) How much can money buy happiness? Is the debate over for the Easterlin Paradox? Radic Stat 98:38–49. http://www.radstats.org.uk/no098/Albor98.pdf

[CR6] Allin P (2007). Measuring societal wellbeing. Econ Lab Market Rev.

[CR7] Anderson B (2004). Time-stilled space-slowed: how boredom matters. Geoforum.

[CR8] Anderson B (2006). Becoming and being hopeful: towards a theory of affect. Environ Plan D.

[CR9] Atkinson S (2011). Care is needed in moves to measure wellbeing. BMJ.

[CR10] Atkinson S, Joyce KE (2011). The place and practices of wellbeing in local governance. Environ Plan C.

[CR11] Atkinson S, Fuller S, Painter J, Atkinson S, Fuller S, Painter J (2012). Wellbeing and place. Wellbeing and place.

[CR12] Block B, Kissell JL (2001). The dance: essence of embodiment. Theor Med.

[CR13] Clarke M, McGillivray M, Clark M (2006). Assessing well-being using hierarchical needs. Understanding human well-being.

[CR14] Conradson D (2005). Landscape, care and the relational self: therapeutic encounters in rural England. Health Place.

[CR15] Coyne JC, Tennen H (2010). Positive psychology in cancer care: bad science, exaggerated claims, and unproven medicine. Ann Behav Med.

[CR16] Csikszentmihalyi M (1991). Flow: the psychology of optimal experience.

[CR17] De Neve J-E, Oswald AJ (2012). Estimating the influence of life satisfaction and positive affect on later income using sibling fixed effects. Proc Natl Acad Sci USA.

[CR18] Deci EL, Ryan RM (2008). Hedonia, Eudaimonia and well-being: an introduction. J Happiness Stud.

[CR19] Diener E, Lucas R, Schimmack U, Helliwell J (2009). Well-being for public policy.

[CR20] Easterlin RA, David PA, Reder MW (1974). Does economic growth improve the human lot? Some empirical evidence. Nations and households in economic growth: essays in honor of Moses Abramovitz.

[CR21] Easterlin RA (1995). Will raising the incomes of all increase the happiness of all?. J Econ Behav Organ.

[CR22] Ehrenreich B (2009). Smile or die. How positive thinking fooled America and the World.

[CR23] Ereaut G, Whiting R (2008) What do we mean by ‘wellbeing’? And why might it matter? Linguistic Landscapes Research Report no. DCSF-RW073 for the Department for Children, Schools and Families

[CR24] Fisher P (2008). Wellbeing and empowerment: the importance of recognition. Sociol Health Illn.

[CR25] Fleuret S, Atkinson S (2007). Wellbeing, health and geography: a critical review and research agenda. NZ Geographer.

[CR26] Foucault M, Burchell G, Gordon C, Miller P (1991). Governmentality. The foucault effect: studies in governmentality.

[CR27] Fredrickson B (2009). Positivity, groundbreaking research to release your inner optimist and thrive.

[CR28] Gutiérrez KD, Rogoff B (2003). Cultural ways of learning: individual traits or repertoires of practice. Educ Res.

[CR29] Haidt J (2006). The happiness hypothesis: putting ancient wisdom to the test of modern science.

[CR30] Hajer MA (1995). The politics of environmental discourse: ecological modernization and the policy process.

[CR31] Hajer MA (2005). Rebuilding ground zero. The politics of performance. Plan Theory Pract.

[CR32] Hajer M, Versteeg W (2005). A decade of discourse analysis of environmental politics: achievements, challenges, perspectives. J Environ Plan Policy Manag.

[CR33] Hall E (2010). Spaces of wellbeing for people with learning disabilities. Scott Geogr J.

[CR34] Huber M, Knottnerus JA, Green J, van der Horst H, Jadad AR, Kromhout D, Leonard B, Lorig K, Loureiro MI, van der Meer JWM, Schabel P, Smith R, van Weel C, Smid H (2011) How should we define health? BMJ 343:d4163 doi:10.1136/bmj.d4163 10.1136/bmj.d4163 21791490

[CR35] Kesby M (2007). Spatialising participatory approaches: the contribution of geography to a mature debate. Environ Plan A.

[CR36] Kim K, Cho YC (2012). A content analysis of advertising transitions: impact of brand name, persona and appeal. J Bus Econ Res.

[CR37] Layard R (2005) Happiness: lessons from a new science. Penguin (Allen Lane), London

[CR38] Little J (2012). Pampering, well-being and women’s bodies in the therapeutic spaces of the spa. Soc Cultural Geogr.

[CR39] Lloyd S, Chalder T, Rimes KA (2012). Family-focused cognitive behaviour therapy versus psycho-education for adolescents with chronic fatigue syndrome: long-term follow-up of an RCT. Behav Res Ther.

[CR40] Lynch D, Laws KR, McKenna PJ (2010). Cognitive behavioural therapy for major psychiatric disorder: does it really work? A meta-analytical review of well-controlled trials. Psychol Med.

[CR41] McGillivray M, Clarke M (2006). Understanding human well.

[CR42] Miller P, Rose N (2008). Governing the present.

[CR43] Mongia M, Hechtman L (2012). Cognitive behavior therapy for adults with attention-deficit/hyperactivity disorder: a review of recent randomized controlled trials. Curr Psychiatry Rep.

[CR44] Nussbaum MC (2000). Women and human development—the capabilities approach.

[CR45] Panelli R, Tipa G (2009). Beyond foodscapes: considering geographies of indigenous well-being. Health Place.

[CR46] Prilleltensky I (2005). Promoting well-being: time for a paradigm shift in health and human services. Scand J Public Health.

[CR47] Putnam R (2001). Bowling alone.

[CR48] Rath T, Harter JK (2010). Wellbeing: the five essential elements.

[CR49] Ricard M (2003). Happiness. A guide to developing life’s most important skill.

[CR50] Riva M, Curtis S, Atkinson S, Fuller S, Painter J (2012). The significance of material and social contexts for health and wellbeing in rural England. Wellbeing and place.

[CR51] Robertson I, Cooper C (2011). Well-being: productivity and happiness at work.

[CR52] Rose G, Degen M, Dasdas B (2010). More on ‘big things’: building events and feelings. Trans Instit Br Geogr.

[CR53] Ryan RM, Deci EL (2001). On happiness and human potentials: a review of research on hedonic and eudaimonic well-being. Annu Rev Psychol.

[CR54] Ryff CD (1989). Happiness is everything, or is it? Explorations on the meaning of psychological wellbeing. J Pers Soc Psychol.

[CR55] Ryff CD, Singer BH, Love GD (2004). Positive health: connecting well-being with biology. Philos Trans R Soc Lond B.

[CR56] Salovey P, Rothman AJ, Detweiler JB, Steward WT (2000). Emotional states and physical health. Am Psychol.

[CR57] Self A, Thomas J, Randall C (2012). Measuring national well-being: life in the UK, 2012.

[CR58] Seligman MEP (1990) Learned optimism. How to change your mind. Pocket Books, Simon & Schuster, New York

[CR59] Seligman M (2002). Authentic happiness.

[CR60] Seligman M (2011). Flourish: a visionary new understanding of happiness and wellbeing—and how to achieve them.

[CR61] Sen A (1999). Development as freedom.

[CR62] Shaw M (2004). Housing and public health. Annu Rev Public Health.

[CR71] Smith DM (2000) Moral geographies: ethics in a world of difference. Edinburgh University Press, Edinburgh

[CR63] Smyth F (2005). Medical geography: therapeutic places, spaces and networks. Prog Hum Geogr.

[CR64] Sointu E (2005). The rise of an ideal: tracing changing discourses of wellbeing. Sociol Rev.

[CR65] Stevenson B, Wolfers J (2008) Economic growth and subjective well-being: reassessing the Easterlin paradox. National Bureau for Economic Research Working Paper No. 14282. NBER, Massachussetts. http://www.nber.org/papers/w14282

[CR66] Stiglitz J, Sen A, Fitoussi J-P (2009) Report by the commission on the measurement of economic performance and social progress. www.stiglitz-sen-fitoussi.fr

[CR67] Tolia-Kelly DP (2006). Affect—an ethnocentric encounter? Exploring the ‘universalist’ imperative of emotional/affectual geographies. Area.

[CR68] Veenhoven R (2000). The four qualities of life. J Happiness Stud.

[CR69] Wilkinson R, Marmot M (2003). Social determinants of health: the solid facts.

[CR70] Wilkinson R, Pickett K (2009). The spirit level.

